# Is Abdominal Fetal Electrocardiography an Alternative to Doppler Ultrasound for FHR Variability Evaluation?

**DOI:** 10.3389/fphys.2017.00305

**Published:** 2017-05-16

**Authors:** Janusz Jezewski, Janusz Wrobel, Adam Matonia, Krzysztof Horoba, Radek Martinek, Tomasz Kupka, Michal Jezewski

**Affiliations:** ^1^Institute of Medical Technology and Equipment ITAMZabrze, Poland; ^2^Department of Cybernetics and Biomedical Engineering, VSB-Technical University of OstravaOstrava, Czechia; ^3^Institute of Electronics, Silesian University of TechnologyGliwice, Poland

**Keywords:** Doppler ultrasound, fetal electrocardiogram, fetal heart rate analysis, fetal state assessment, fetal outcome

## Abstract

Great expectations are connected with application of indirect fetal electrocardiography (FECG), especially for home telemonitoring of pregnancy. Evaluation of fetal heart rate (FHR) variability, when determined from FECG, uses the same criteria as for FHR signal acquired classically—through ultrasound Doppler method (US). Therefore, the equivalence of those two methods has to be confirmed, both in terms of recognizing classical FHR patterns: baseline, accelerations/decelerations (A/D), long-term variability (LTV), as well as evaluating the FHR variability with beat-to-beat accuracy—short-term variability (STV). The research material consisted of recordings collected from 60 patients in physiological and complicated pregnancy. The FHR signals of at least 30 min duration were acquired dually, using two systems for fetal and maternal monitoring, based on US and FECG methods. Recordings were retrospectively divided into normal (41) and abnormal (19) fetal outcome. The complex process of data synchronization and validation was performed. Obtained low level of the signal loss (4.5% for US and 1.8% for FECG method) enabled to perform both direct comparison of FHR signals, as well as indirect one—by using clinically relevant parameters. Direct comparison showed that there is no measurement bias between the acquisition methods, whereas the mean absolute difference, important for both visual and computer-aided signal analysis, was equal to 1.2 bpm. Such low differences do not affect the visual assessment of the FHR signal. However, in the indirect comparison the inconsistencies of several percent were noted. This mainly affects the acceleration (7.8%) and particularly deceleration (54%) patterns. In the signals acquired using the electrocardiography the obtained STV and LTV indices have shown significant overestimation by 10 and 50% respectively. It also turned out, that ability of clinical parameters to distinguish between normal and abnormal groups do not depend on the acquisition method. The obtained results prove that the abdominal FECG, considered as an alternative to the ultrasound approach, does not change the interpretation of the FHR signal, which was confirmed during both visual assessment and automated analysis.

## Introduction

Fetal heart activity is a primary source of information which enables assessment of the fetal state during pregnancy and at labor. This information is obtained mainly through analysis of the fetal heart rate (FHR) signal being formed from the instantaneous values calculated according to the formula: FHR [bpm] = 60000/T [ms]. The FHR values are expressed in beats per minute, and T is the time interval between two consecutive fetal heart beats that comprises one complete cardiac cycle. Together with additional signals describing the uterine contractile activity and fetal movement profile, the FHR signal constitutes the cardiotocographic record. Acquisition of these additional signals is quite simple, but measurement of the fetal heart rate has been always a challenge. Already in 1960s the fetal electrocardiogram was recorded for the first time by means of electrode attached to fetal head. The quality of such recorded direct fetal electrocardiogram (FECG) is usually very good, and thus it enables, using a quite simple processing method, determination of the beat-to-beat intervals with very high accuracy. However, the invasive approach and application limited to the labor only caused that direct method did not found wide application in clinical practice.

As a result of further research and development the noninvasive ultrasound (US) method has become a standard approach since early 1970s, as it can be used both during pregnancy and labor. At present, all bedside fetal monitors intended to use in clinical conditions are based on the pulsed Doppler ultrasound technique, with measurement transducer attached to maternal abdomen. Principle of operation relies on internal processing of the envelope of the US beam reflected from moving parts of fetal hearts—valves or walls, to find the episodes corresponding to consecutive heart beats. However, a complex structure and varying content of the US signal, usually caused by relocation of the fetal heart in relation to a transducer during monitoring session, make a determination of the beat-to-beat interval very difficult (Khandoker et al., 2009; Marzbanrad et al., 2014). Therefore, a correlation techniques, considering full shape of the analyzed signal, have been applied. The cross-correlation technique with changeable template appeared to be too sensitive to US signal changes, which resulted in considerable signals loss. Thus, an autocorrelation function with adaptive window selection has been applied in next generation fetal monitors. However, the autocorrelation function does not detect the consecutive heart beats but only determines the instantaneous periodicity of the US signal envelope which corresponds to cardiac cycle being measured. This leads to effect of averaging of neighboring cardiac cycles and thus decreasing of FHR determination accuracy in relation to fetal electrocardiography (Lee et al., 2009; Voicu et al., 2010). The obtained FHR signal is provided by the bedside monitor as the trace in a printout with established time scale of 1, 2, or 3 cm/min. As long as the FHR trace has been analyzed visually, the lower accuracy did not affect significantly the fetal state assessment. More important was to ensure the trace continuity which allowed clinicians to observe a general tendency of the fetal heart rate changes, and to recognize the features representing longitudinal FHR patterns relating to the fetal state, like acceleration or deceleration. It was found that the evaluation of fetal state, when based on visual interpretation, has been mainly affected by low inter- and intra-observer agreement (Jezewski et al., 2002; Romano et al., 2016a). That was a result of both complexity of the FHR signal and the fact that important part of information relating to instantaneous changes of FHR values has been hidden from a naked eye. These changes are considered to be very important FHR characteristics, reflecting appropriate neurological modulation of the FHR.

Thus, further development stage of fetal monitoring was aimed at automated analysis of the FHR signal and its implementation as built-in procedure of bedside monitors as well as in computer-aided fetal monitoring system. Some other requirements important for monitoring the pregnant women in hospital, like surveillance of many patients, detecting and alerting of symptoms of fetal distress, or electronic archive with the signals and perinatal data, have made the computer-aided system with online automated analysis the standard in modern obstetrics (Wrobel et al., 2013, 2015a). Automated analysis comprises detection and description of the above mentioned FHR features, like acceleration and others, as well as determination of the instantaneous FHR changes by providing a set of indices to evaluate the long-term and short-term (beat-to-beat level) variability of the fetal heart rate.

Automated online analysis provides a quantitative description of the FHR, but the final interpretation of the record is still done by a clinician. There are a number of papers relating to automated classification of the FHR recordings by using different methods of computational intelligence like neural network, support vector machines or epsilon-insensitive learning (Czabanski et al., 2008, 2013). However, taking into account that the input data set comprised the automatically determined features of the FHR signals, collected from the clinical databases (Chudacek et al., 2014), the obtained classification results should be faced to the limitations of the ultrasound approach as it is discussed below (Voicu et al., 2014; Wrobel et al., 2015c).

The variability indices were originally defined using the beat-to-beat intervals determined from the direct electrocardiogram. Their straight application to the FHR signals being provided by fetal monitors raised a question how a limited accuracy of the ultrasound approach affects determination of the cardiac intervals, and thus the variability indices values. Several research studies were aimed at evaluation of the reliability of the ultrasound method in reference to the direct electrocardiography (Ibrahimy et al., 2003; Reinhard et al., 2010, 2012; Cohen et al., 2012; Kimura et al., 2012; Desai et al., 2013; Kording et al., 2015). However, they were aimed at comparing the signal loss episodes or directly the FHR values. In our study we showed that the error of cardiac cycle determination (instantaneous FHR value) has not been correlated with the FHR variability indices error. It means that the measurement accuracy resulting from the fetal monitor specification cannot be directly related to the results of the computer-aided analysis of the FHR variability. In general, we concluded that modern fetal monitors using the Doppler US technique are not able to provide the signal with the accuracy required for reliable quantitative evaluation of instantaneous FHR variability, particularly the short-term variability, based on the indices calculated automatically (Voicu et al., 2014). Fortunately, the values of indices determined in that way are underestimated, which prevents the fetal distress signs from being undetected.

Several attempts were carried out to improve the reliability of the ultrasound method by using advanced signal processing of the Doppler envelope (Jezewski et al., 2011), but none of them have been applied in the bedside monitors yet. In Wrobel et al. (2008) the method has been proposed to improve the reliability of the FHR variability indices, which relies on the errors recognized in the ultrasound measurement channel.

The fact, that the FHR signal obtained from the ultrasound approach has been recognized as not good enough to fully exploit the potential of automated analysis offered by computer-aided fetal monitoring system, brought back an interest of the fetal electrocardiography (Fuchs, 2014). However, taking into account the need to monitor a whole pregnancy period, only a noninvasive approach could be considered, which relies on indirect recording the FECG from electrodes located on maternal abdominal wall (Ungureanu et al., 2009; Vullings et al., 2010; Kimura et al., 2012; Khalaf et al., 2013; Behar et al., 2014; Agostinelli et al., 2015b).

Another important issue for development of effective abdominal electrocardiography refers to a growing interest in high-risk pregnancy telemonitoring at home (Wrobel et al., 2015b). When using the ultrasound-based fetal monitor the transducer has to be carefully placed to ensure the ultrasound beam is focused on the fetal heart. What's more, during monitoring session the transducer may require repositioning due to a change of fetus position. Otherwise, the signal loss occurs which may cause, in case when a woman performs the monitoring session alone, her unfounded fear and unpredictable reaction. When the abdominal electrodes are fixed on the abdomen, the patient can easily verify the signal loss, which in that case occurs only when one of the electrodes peels off (Karvounis et al., 2007; Kolomeyets and Roshchevskaya, 2013; Agostinelli et al., 2015b).

Improvement of the measurement instrumentation, electrode technology and the signal processing methods that have been noticed during recent years, enabled to cope with the problems connected with development of the abdominal fetal electrocardiography (Kotas, 2008; Vullings et al., 2010). The signal acquired from fetus head is in fact “pure” fetal electrocardiogram, whereas the abdominal signal includes also the maternal electrocardiogram (MECG) and some noise coming mainly from muscle activity (Taralunga et al., 2009, 2014; Martinek et al., 2016). Thus, the crucial step in extraction of the FECG from the abdominal signal is a suppression of maternal electrocardiogram while preserving the fetal QRS complexes (Melillo et al., 2014; Agostinelli et al., 2015a). The energy of MECG is many times higher than the energy of FECG, and what's more the frequency band of both these components partly overlaps which makes simple filtering useless (Karvounis et al., 2007). A number of different approaches to MECG suppression and detection of fetal QRS complexes were presented in literature (Ungureanu et al., 2007; Liu and Luan, 2015; Poian Da et al., 2016). The system for acquisition of abdominal signals and original method for FECG extraction were proposed by the authors, and the indirect fetal electrocardiography was evaluated in relation to the gold standard—direct FECG approach (Jezewski et al., 2012). Referring to the results obtained in our previous study, concerning a comparison of ultrasound approach with the direct FECG (Jezewski et al., 2006), we concluded that the abdominal fetal electrocardiography provides accuracy not worse than the ultrasound method does. However, in all studies where the US method or abdominal FECG was compared with direct FECG, the results were obtained only for the signals being acquired during labor. Considering that fetal development, taking place during a whole pregnancy period, affects the characteristics of the FHR signals, we decided to carry out the comparison of the abdominal FECG and ultrasound method based on the signals collected during pregnancy. It is obvious that such approach excludes the direct electrocardiography from the study, and causes some problems for comparison methodology due to a lack of reference data (Sato et al., 2011; Cohen et al., 2012; Kimura et al., 2012). Since both types of the signals were acquired by means of two sets of instrumentation, another important problem has been recognized—the FHR signals synchronization, i.e., finding the corresponding cardiac cycles. It should be noticed that in case of ultrasound-based monitor, the FHR signal is provided through its output only as the measurement values of instantaneous heart rate evenly spaced with 250 ms. On the other hand, the system for noninvasive FECG is able to provide, along with the evenly spaced signal, the time event series with durations of consecutive cardiac cycles.

In this work the methodology is proposed to compare two different methods for fetal heart rate monitoring. Its originality relates to the fact that comparison has been carried out not only in relation to the corresponding cardiac cycle values, but also to the clinically important indices describing the instantaneous FHR variability.

## Methods

The research material comprised the FHR signals acquired simultaneously using the Doppler ultrasound as well as the electrocardiographic methods in a group of 70 pregnant women. From a number of monitoring sessions performed for each patient, we selected only one recording acquired around 1 week before delivery, with a length of at least 30 min (Georgieva et al., 2014). All the recordings are accompanied by information on fetal outcome: gestational age at birth, blood gas parameters pH and BE, percentile of fetal birth weight, Apgar score, information about a possible stay in the NICU. The patients were monitored by simultaneously using two popular in maternity wards, systems for fetal and maternal monitoring: MONAKO and KOMPOREL. Unfortunately, these systems were unable to synchronize recorded signals during the monitoring session. The time shift between signals beginnings in each session could reach up to a few minutes, whereas in case of comparative studies the precise synchronization is required (even on the level of individual heartbeats). Hence, the problem of signals synchronization has been considered as a significant challenge.

As the result of each simultaneous monitoring session, two files were obtained of the native format, where the FHR signal is represented by the values measured evenly with 250 ms period. Files from the MONAKO System comprise the FHR_U signal captured from the output of fetal monitor equipped with the ultrasound transducer (Hewlett-Packard M1351). The files from the KOMPOREL System provide the FHR_E signal being determined on a basis of fetal electrocardiogram recorded from the abdominal wall of the mother. The fetal electrocardiogram is recorded by using four electrodes placed on the maternal abdomen. The crucial step in extraction of the FECG from the abdominal signal is a suppression of maternal electrocardiogram while preserving the fetal QRS complexes (Castilloa et al., 2013; Martinek et al., 2015). The proposed method for the MECG suppression is based on subtracting the pattern of maternal P-QRS-T complexes and spatial filtering. It ensures correct determination of the fiducial points as well as the factors scaling the pattern. The algorithm for detection of the fetal QRS complexes is based on a matched filtering approach in order to reduce the sensitivity to interferences. Additionally, the detection is carried out with a set of decision rules to predict the duration of the next beat-to-beat cardiac cycle (Matonia et al., 2006). As a result of the FECG analysis, the time event series is obtained—as the time markers when the successive fetal heartbeats were detected—the QRS complexes, which is then used to determine the FHR_E signal as the values with 250 ms period (Guerrero Martinez et al., 2006; Almeida et al., 2013, 2014).

### Signals synchronization

The procedure for synchronization of each pair of the FHR_U and FHR_E signals consisted of two stages. In the first stage an initial visual adjustment was supported by a dedicated program for visualization of the signals. This program as well as all the others, created for the purpose of this work, was developed in LabView environment (NationaI Instruments) (Desai et al., 2013). After coarse synchronization of the signals the common part of FHR_U and FHR_E signals was separated. It relied on moving the beginning and end of one signal, to indicate the fragment of interest according to the other signal. At this stage the signals quality had to be good enough to allow recognizing the characteristic features common for both signals, and constituting the so called centering points, and the common part of the signals had to have non-zero length. These conditions were not met in case of three recordings, hence in further processing only the set of 67 patients were included.

**Figure 1:**
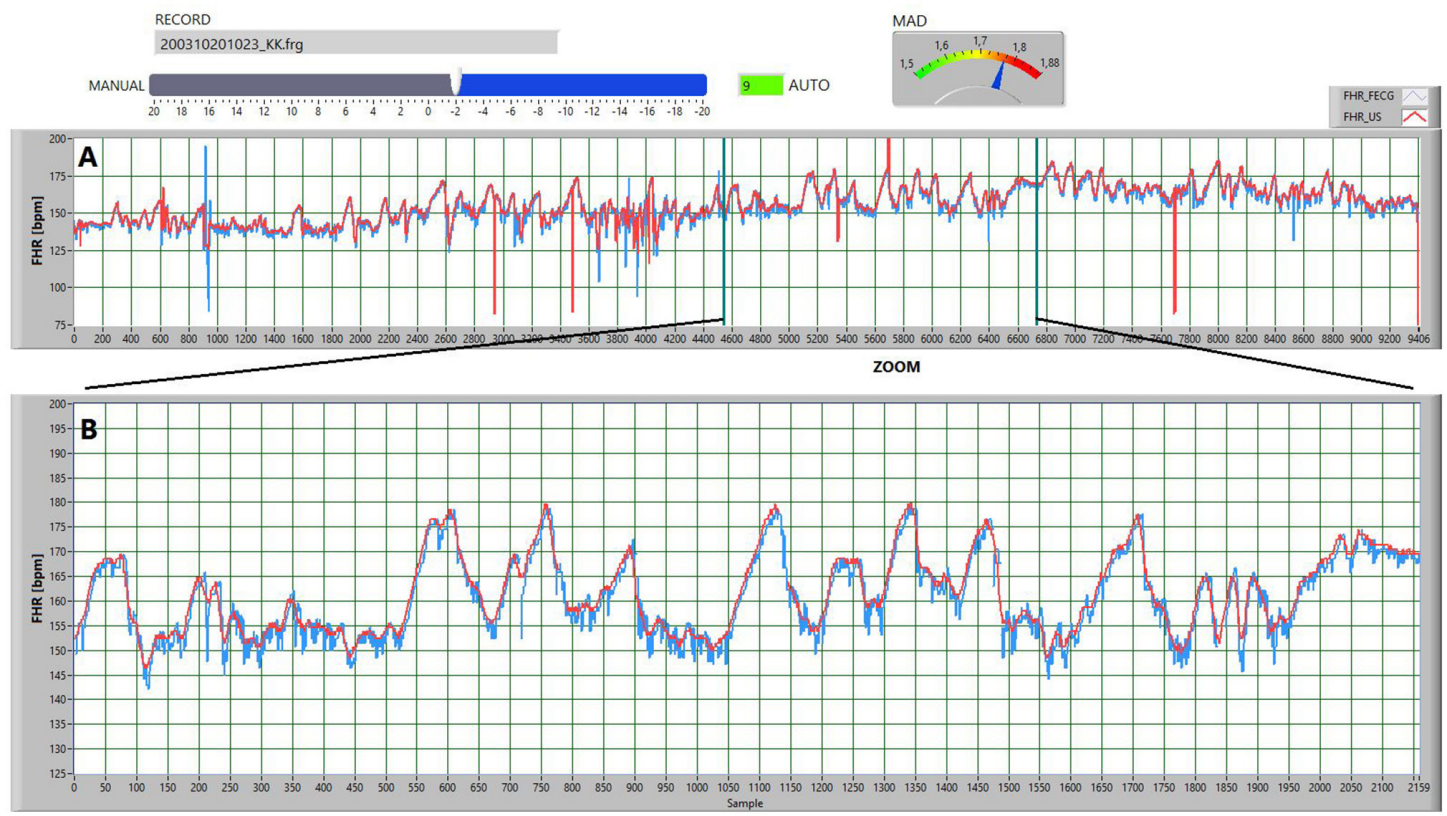
**The screen illustrating the operation of the procedure for synchronizing the two signals, determined via the ultrasound (red FHR_U) and electrocardiographic (blue FHR_E) methods. (A)** Presents the concept of automated synchronization via minimizing the mean error. The current value of the mean absolute difference MAD parameter is displayed on the gauge. Auto mode had automatically set up the value of the shift parameter to 9 samples forward. However, basing on a visual analysis of the signals, that shift was manually corrected using a slider—the FHR_U signal was shifted by 2 samples backward in relation to the FHR_E signal. **(B)** Shows the enlarged signal fragment from part A, but after a procedure for removing the sudden FHR changes. It allows for additional manual synchronization and final validation of the recording for further investigations.

In the second stage the signal validation was conducted, as well as precise synchronization of both signals, at the level of individual FHR values provided every 250 ms. The developed software enabled semi-automated synchronization. The program automatically found the time shift between the signals to ensure the minimum differences between the corresponding values, which mostly led to proper synchronization. After that, the visual verification was carried out with a possibility of additional time shift correction, followed by the final acceptance (Figure 1).

The software for determination of the optimal time shift between the analyzed signals was using the synchronization function based on the mean absolute differences (MAD), determined for the corresponding (applying the time shift) FHR_U and FHR_E values. To improve the performance of synchronization function it was necessary to further reduce the influence of random interferences appearing in the FHR signals, as well as sudden value changes resulting from the measurement errors or potential acceleration and deceleration episodes. Hence, the segments with sudden changes in the FHR signal were excluded from the function determination, if the absolute difference between a given value and the preceding one was higher than 10 bpm (Spilka et al., 2012). If a given FHR value was rejected, the next one was compared to the mean calculated from the previous values (including the rejected) in the 240 values window. The FHR values were also rejected from the signal, which were suspected to represent the maternal heart rate—the details of the algorithm are presented in Wrobel et al. (2015b). This type of erroneous measurements occurs in the FHR_U signal, as it is typical for the ultrasound method and quite frequent in the US-based fetal monitors. The above-mentioned preprocessing is only intended to synchronize the signals and do not change their information content.

The optimum time shift, corresponding to the fully synchronized signals, was obtained for the function minimum, when applying additional shift in the range from −25 to +25 FHR values (measured every 250 ms), in respect to the signals synchronized after the first stage. If at the beginning or end of a given signal any interference associated with the start or end of the monitoring session occurred, they were also removed in the trimming process. Trimming to the full minutes in turn, results from the fact that the analysis of the instantaneous FHR variability is always carried out within a 1-min signal segments. Finally, as a result of the synchronization procedure some signal pairs could be shortened by as much as 4 min. After the second stage of synchronization the common part of the analyzed signals is trimmed to the largest whole number of the minutes (the number of FHR values was a multiple of 240).

We assumed that the minimum length of synchronized signals (constituting the pair) subjected to further analysis should be 10 min. According to that criterion only one recording was rejected, and 66 recordings were left.

### Signal loss analysis

The next processing step consisted of verifying the signal pairs in terms of their quality, measured both by a size and nature of the signal loss episodes. Episodes of signal loss are preliminarily detected by the monitoring systems used, and represented by zero values in the FHR signal. For the purpose of this work also the potential erroneous FHR values, as not meeting the adopted criteria, were marked as signal loss episodes, using the dedicated developed program. Signal segments were considered to be signal loss if they did not meet the van Geijn modified criterion (van Geijn, 1980), proposed in Jezewski et al. (2012). That was applied to these FHR values, which were considered as errors by a procedure for sudden changes removal used in the second stage of synchronization. In this case, the established thresholds are: the absolute difference between a given FHR value and the preceding one greater than 20 bpm, and window width equals to 100 values. The segments, suspected to contain the maternal heart rate signal, were indicated as the signal loss episodes—similarly as in the second stage of synchronization. Finally, as a result of the signal loss analysis, the FHR_U and FHR_E signals were obtained with additional information about detected gaps (FHR values equal to zero). The signal loss level is defined as a percentage of the duration of signal loss episodes (the number of FHR values equal to zero) in relation to the total duration of the signal (all FHR values). Taking into account the maximum level of signal loss of 30% in either FHR_U or FHR_E signal, five recordings were removed. Additionally, in terms of uniformity of signal loss distribution in time, four questionable recordings with signal loss between 20 and 30% were visually assessed. Only one recording was excluded due to the accumulation of the signal loss (equal to 23%) in the middle part of the FHR_U signal. The final research material consisted of signal pairs from 60 monitoring session. The total length of recordings was equal to 1995 min. The length of individual recordings varied from 11 to 64 min, with an average of 33.3 min.

### Direct signals comparison

For the final set of recordings, consisting of the FHR_U and FHR_E signal pairs, some descriptive statistics of signal comparison were calculated, both on a global basis as well as at the level of particular FHR values. These statistics include (calculated for individual recordings): the recording duration, signal loss level, mean value of the differences between the corresponding instantaneous FHR values (MD), standard deviation (SD), mean absolute difference (MAD), as well as the summary statistics for the entire research material. The difference between the pairs of corresponding instantaneous values of FHR_U and FHR_E was expressed dually: as the heart beats per minute (bpm) as well as in milliseconds. The second representation is obtained by conversion of the FHR values into intervals between successive heart beats, according to the hyperbolic transformation with the 60,000 factor.

As equally important it is assumed the comparison of the signals in a format commonly used in automated analysis of the low variability FHR signal components (Jezewski et al., 2002). In this format the successive values of the signal are determined by averaging 10 consecutive original FHR values (time series measured every 250 ms). Important part in the averaging process is the removal of the signal loss episodes, marked as zero values. If, while averaging 10 original FHR values, more than four values are marked as signal loss, the resulting value of 2.5 s period is also considered as signal loss and is assigned with zero value.

### Indirect signal comparison via clinical parameters

As shown in the literature (Jezewski et al., 2016), the differences from the direct comparison of signals are often not correlated with differences in values of clinically important parameters of quantitative description of FHR signal, determined by the fetal monitoring systems (Georgieva et al., 2012). These parameters are used by clinicians, interpreting the FHR signals in order to assess the fetal state. Therefore, it was considered as important to identify the impact of the FHR signal acquisition method on the clinically significant parameters. For that purpose, the synchronized FHR_U and FHR_E signals were saved into the native format files (measured with 250 ms) and reloaded to the archive of MONAKO System. Thanks to an option of reanalysis of the archival records, for each of 60 recordings (120 FHR signals), the quantitative parameters describing the variability patterns detected in the FHR signal were determined automatically. As the result, the lists of parameters were obtained for the FHR_U and FHR_E signals. They included the parameters describing some patterns of FHR variability in the time domain: mean value of FHR (M_FHR), mean value of the FHR baseline (M_BL), number of detected acceleration (ACC) and deceleration (DEC) episodes (Georgieva et al., 2012; Wrobel et al., 2013). Additionally, an assessment of the instantaneous FHR variability was provided as: duration and value of the high (HE_D and HE_V) and low (LE_D and LE_V) variability episodes, average value of the long-term variability (LTV) and short-term variability (STV) indices, as well as the FHR oscillations (OSC) together with the percentage of different oscillation types (OSC_I÷OSC_IV) (Jezewski et al., 2016). Inconsistencies of the above parameters calculated for the corresponding FHR_U and FHR_E signals were estimated using the symmetric mean percentage difference SMPD, where the differences between values are related to their mean. Since the normality assumption was verified using the Shapiro-Wilk test, the statistical significance (using paired Student's *t*-test) of the differences between the corresponding parameters obtained in the FHR_U and FHR_E signals was examined.

### Indirect signals comparison via beat-to-beat variability

It is generally believed that a very high predictive value in relation to the early detection of the fetal distress is provided by the instantaneous FHR variability parameters (Cesarelli et al., 2009). They are determined from the FHR signal in a form of the time event series—a sequence of events unevenly located in time, providing the successive cardiac cycles duration expressed in milliseconds.

This format significantly differs from that available at the output of a fetal monitor—the FHR values evenly spaced at every 250 ms. This measurement period has been established to be not longer than the shortest physiologically allowed heart cycle, however with characteristic information redundancy for low FHR values (e.g., the FHR value equal to 50 bpm is represented by four duplicated subsequent values; Lee et al., 2009; Goncalves et al., 2013).

Therefore, the FHR_U and FHR_E signals were subjected to reconstruction of the above mentioned time event series representation. This procedure relied on taking from the evenly distributed time series, the values according to the timing signal being constituted by the fetal QRS complexes additionally obtained from the KOMPOREL System. The resulting signal is a sequence of time-ordered events corresponding to subsequent occurrences of the fetal QRS complexes (or more precisely the R-waves). Created according to the described procedure the FHR_U and FHR_E signals in the form of time event series, provided the basis values for determination of the instantaneous variability indices. These indices, widely acclaimed in the literature (Romano et al., 2016b), quantitatively describe the long- and short-term FHR variability.

In this study the following indices were analyzed: Haan_LTI, Haan_STI, Yeh_II, Yeh_STI, Organ_LTV, Organ_STV, Dalton_LTV, Dalton_STV, Zugaib_LTV, Zugaib_STV. In order to standardize the results the indices were determined within 1-min segments (Kubo et al., 1987; Jezewski et al., 2006). Each consecutive segment comprised only those instantaneous FHR values which were determined using the heart beats contained in the given segment (Cesarelli et al., 2009).

While processing a given segment, if percentage of valid values, relevant for a given index (according to its definition), was less than 20%, it was assumed that the index value was undetermined for that minute. Such cases are the result of the signal loss episodes in the analyzed signals. In a series of minute values calculated for a given signal, they are defined as a 1-min loss of the given index value and marked with the value of −1. The index average value for a given signal is calculated from all 1-min values, excluding those marked as undetermined. Additionally, a non-linear parameter of instantaneous FHR variability was proposed, in a form of the regularity measure—the sample entropy index (SampEn) (Signorini and Magenes, 2014). It was determined in the windows covering 300 heart events (FHR values), and expressed in milliseconds as a measure of period. The parameters of SampEn function were set at: dim = 1 and *r* = 0.1. For the given signal, the SampEn index represents the mean value of sample entropy determined in successive windows. Inconsistencies of indices describing the instantaneous FHR variability, determined for corresponding FHR_U and FHR_E signals were evaluated with the SMPD, where the difference between values are related to their mean. The statistical significance (using paired Student's *t*-test) of the differences between the values of the indices obtained for each signal pair was also examined.

### Indirect signals comparison via fetal outcome prediction

Analysis of the FHR signal leads to its classification as corresponding to normal or abnormal fetal state. Since at the time of fetal monitoring, there is no other diagnostic method which could be able to confirm a correctness of the signal classification, the FHR signals, being acquired during pregnancy, are retrospectively assigned to true fetal outcome (newborn state) (Chudacek et al., 2014; Romano et al., 2016a). It is justified, as in obstetrics it is assumed that the normal fetal outcome has to be result of proper fetal development during the pregnancy period. Excluding the cases when the labor process itself caused negative effects for the fetal state, the same assumption can be applied for abnormal fetal outcome. It is generally believed that this relationship is maintained in case of deliveries by cesarean section due to the maternal reasons. In the collected database the vast majority of cases were of this type. The 60 patients (recordings) were classified as belonging to a normal or abnormal group using the information on fetal outcome. The abnormal state was set if at least one of the following conditions was met: Apgar score (at 5 min) <7, pH <7.2, BE >12, NICU stay > 24 h, or birth weight percentile <5%. Finally, the research material included 19 recordings with abnormal state and 41 with normal state assigned retrospectively. It is important that for the majority of patients with abnormal fetal outcome, the pregnancy was ended by cesarean section due to the maternal indications (16 cases). It suggests that course of delivery imposed no negative effect on the newborn. The database contains six recordings for which the abnormal state was set due to three or more conditions met.

The analysis of ability for prediction of the fetal outcome was performed separately for the ultrasound and the fetal electrocardiography approaches. Each FHR_U (FHR_E) signal was represented by the feature set comprising: 15 parameters determined by the MONAKO System, 10 indices describing the instantaneous FHR variability and SampEn entropy measure. The differences between the values of these features obtained in two groups (normal and abnormal fetal outcome) were expressed by the mean percentage difference (MPD) for both groups—where the normal outcome group was taken as reference. The statistical significance of the difference between the mean values of each feature obtained for two groups was assessed using Student's *t*-test.

The indirect comparison of FHR_U and FHR_E signals, as for predicting the fetal outcome, was based on the capability to classify the FHR signals from a given acquisition methods into normal and abnormal analyzing the determined clinical FHR parameters.

## Results

Comparison analysis considering two different methods of FHR signal acquisition has been carried out using 60 pairs of FHR_E and FHR_U signals, which were obtained during the monitoring sessions of 60 patients. After a full signals synchronization and trimming, the total length of recordings was equal to 1995 min, with an average length of 33.3 min (*SD* = 10.9 min). The recordings were characterized by low signal loss (details in Table 1). For the US method the mean signal loss was equal to 4.5%, whereas for the FHR signals obtained via FECG, the loss level was more than two times lower, which is expressed by the mean signal loss of only 1.8%. High signal loss was observed in few recordings, especially for FHR_U. It could be noted that for more than half of the recordings, the signal loss for both methods did not exceed 1%. Such low level of the signal loss and the sufficient length of individual recordings enabled further estimation of the inconsistency between both acquisition methods, using the mean values of clinical quantitative parameters, determined during the FHR signal analysis.

**Table 1:** **Descriptive statistics of the signal loss and values of differences MD, MAD, determined for the final set of recordings from the electrocardiography (FECG) and the ultrasound method (US), where the FHR signals were expressed as the original 250 ms measures, and as the values averaged over 2.5 s periods**.

	**FHR 250 ms**						**FHR 2.5 s**			
	**Signal loss (%)**		**FECG-US (bpm)**		**FECG-US (ms)**		**Signal loss (%)**		**FECG-US (bpm)**	
	**FECG**	**US**	**MD**	**MAD**	**MD**	**MAD**	**FECG**	**US**	**MD**	**MAD**
Mean	1.80	4.53	−0.23	1.24	0.71	3.83	1.35	4.29	−0.09	0.71
SD	3.01	6.52	0.38	0.46	1.25	1.62	2.59	6.42	0.40	0.43
Median	0.60	0.95	−0.19	1.12	0.59	3.36	0.35	0.75	−0.05	0.58
Min	0.00	0.10	−1.29	0.50	−4.52	1.39	0.00	0.00	−1.50	0.23
Max	14.70	26.80	1.54	2.86	5.33	9.91	12.50	26.40	1.49	2.50

**Figure 2:**
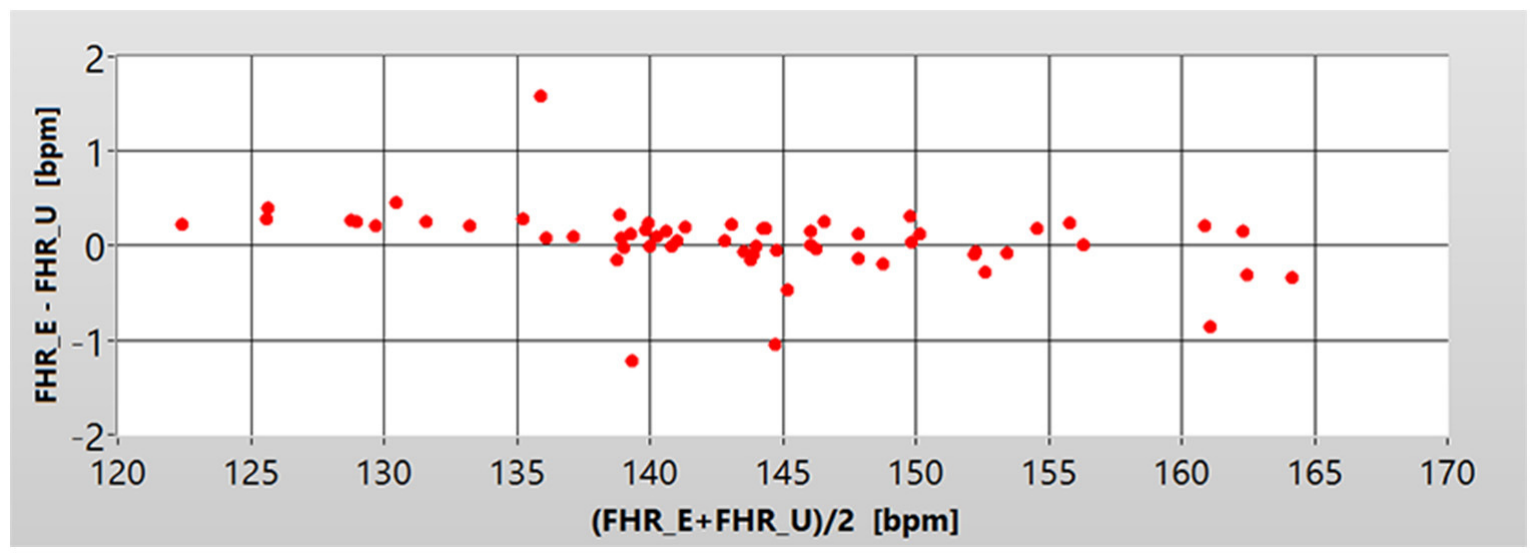
**Bland Altman plot showing the dependence of the mean value of the differences MD (Y axis) between corresponding instantaneous values of FHR_U and FHR_E, in relation to the average fetal heart rate in the recording (X axis), for each of the 60 pairs of signals**. The values are expressed in beats per minute—bpm.

A direct comparison of the FHR_E and FHR_U signals has been based on estimation of the differences between the corresponding instantaneous FHR values (provided every 250 ms). It enabled the metrological assessment of the inconsistency between the two acquisition methods. Comparison at that stage was performed for each individual recording and the summary of descriptive statistics for entire research material is presented in Table 1. The mean difference value (MD) obtained for all recordings was −0.23 bpm, which in relation to an average value of FHR (about 140 bpm) gives a relative error of 0.2%. It means that the measurement bias between the two methods does not occur. When analyzing the MD values calculated for particular recordings we noted that it did not depend on the measured FHR signal value. It is shown by the Bland-Altman plot of MD values against the average FHR values (from FHR measured at 250 ms) obtained for particular recordings (Figure 2). From the point of view of both visual and automated assessment of the FHR signal variability, the more important seems to be the MAD, which was equal to 1.24 bpm for all the considered signals. Such value does not affect a visual evaluation of the signal, since it is lower than the printing resolution of the FHR waveforms, as well as the resolution of a human eye.

In the computer-aided system, the automated analysis aimed at determination of clinically important FHR patterns is carried out using the FHR values averaged over 2.5 s. It makes the comparison between FHR_E and FHR_U signals represented by such averaged values especially important. Averaging processes caused a slight decrease of the signal loss in both types of signals, to the values: 4.3% in US, and 1.4% in FECG (Table 1). Comparison at this stage was performed for particular recordings and the summary of descriptive statistics for entire research material is presented in Table 1. In general, inconsistency between FHR signals after averaging decreased. In this case, the bias between the two methods also does not occur. The MAD value has decreased significantly, to only 0.7 bpm, which is below the 1 bpm level—the minimum accuracy of the FHR measurement. In relation to the average value of the FHR signal (140 bpm), the relative inconsistency was equal to 0.5%. Considering these results we could assume that such small inconsistency should lead to the similar values of clinical parameters provided by both methods. However, we have to keep in mind that some of these parameters are particularly sensitive to the FHR changes, being a result not only of the mean FHR difference, but rather of the distribution of FHR differences in time. In contrast, some other parameters of the FHR signal are sensitive to temporary high differences in the FHR signals. So, in case of these parameters the differences between both methods may occur. Such formulated assumptions have been verified in the next stage of the inconsistency analysis—the indirect comparison of both signals. It was based on the interpretation of the differences between the particular FHR signal parameters, which are provided by an automated analysis in the fetal monitoring system.

**Figure 3:**
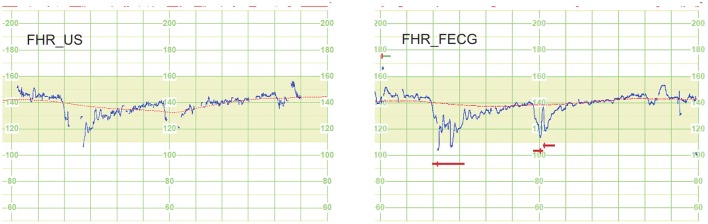
**An example of a 12-min fragment of signal pair—result of an indirect comparison to evaluate the impact of the FHR signal acquisition method on clinically relevant parameters, determined by a computer-aided fetal monitoring system**. The autocorrelation technique, commonly used in the US method to determine the signal periodicity, is often not able to follow the rapid decrease of FHR_U signal related to deceleration, which results in signal loss episodes. This, in turn, causes that the deceleration is not recognized, because it does not meet the established criteria of amplitude and duration. Graphic markers of the analysis results illustrate the signal loss (above the curve), the estimated FHR baseline (line fitted on FHR curve) and detected deceleration episodes (horizontal bars under the curve).

Descriptive statistics (mean values and SD) for individual parameters describing quantitatively the FHR signal, which have been determined for signals from both methods, are presented in Table 2. For each parameter the differences between the FHR_E and FHR_U signals, were assessed using the SMPD. It was justified because for any of those parameters no significant difference between them was noted. As it could be expected, the low SMPD value of 0.1% obtained for M_FHR and M_BL parameters (being significantly dependent on an averaging process of the FHR measurements), was similar to the relative inconsistency reported in the direct signals comparison. In turn, the SMPD values calculated for the following parameters: HE_D, LE_D, STV, ACC, DEC, OSC_IV, differ significantly from the values reported for other parameters, and even more from the results of the direct comparison. It confirms the above mentioned assumption that some parameters (e.g., STV) are sensitive to distribution of the FHR differences in time, whereas another ones (e.g., DEC) to a temporary high difference value. Particularly, high SMPD = 54% noted for the number of recognized decelerations, is a results of two factors: the direct differences between the signal values and the differences in the signal loss episodes. The signal loss for FHR_U is on average twice higher than for FHR_E. In addition, the autocorrelation technique, commonly used in the US method to determine the signal periodicity, is often not able to follow the rapid decrease of FHR signal related to deceleration, which results in signal loss episodes (Figure 3). This, in turn, causes that the deceleration is not recognized, because it does not meet the established criteria of amplitude and duration.

**Table 2:** **Values of clinically important parameters of quantitative description of FHR-E and FHR-U signals, from FECG and US, obtained in a computer-aided fetal monitoring system, together with the symmetric mean percentage difference SMPD estimating the inconsistencies between both the methods**.

**Parameters**	**FHR_E**		**FHR_U**		**SMPD (%)**
	**Mean**	**SD**	**Mean**	**SD**	
M_FHR (bpm)	143.19	9.35	143.32	9.49	−0.1
M_BL (bpm)	141.70	9.55	141.79	9.60	−0.1
ACC (number)	6.62	5.31	6.12	5.29	7.8
DEC (number)	0.40	0.87	0.23	0.56	54.0
LTV (ms)	39.54	11.90	37.91	11.40	4.2
STV^*^ (ms)	6.35	2.41	5.68	2.02	11.1
HE_D (min)	12.72	12.15	10.60	11.49	18.2
LE_D (min)	7.85	8.01	8.72	7.91	−10.5
HE_V (ms)	53.59	9.67	54.99	7.81	−2.6
LE_V (ms)	18.67	3.22	18.78	3.39	−0.6
OSC (ms)	13.40	3.80	12.91	3.70	3.7
OSC_I (%)	10.51	14.39	11.05	15.30	−5.0
OSC_II (%)	27.77	15.59	29.29	15.39	−5.3
OSC_III (%)	45.31	18.19	45.48	19.11	−0.4
OSC_IV (%)	8.96	9.25	7.81	9.06	13.7

* *p < 0.05 (paired t-test)*.

Indirect comparison of the FHR_U and FHR_E signals was performed on the basis of the variability indices defined for signal represented as time event series—the heart beats. Summary of the results (mean values, SD, and SMPD) for the selected 11 parameters describing the FHR signal variability is presented in Table 3. The results clearly show that the FHR_E signal is characterized by higher variability then the FHR_U. Inconsistencies for the long-term variability indices were at about 10%, whereas for the short-term indices they were five times higher, reaching about 50%. For the short-term variability we noticed significant difference for both methods.

**Table 3:** **Results of the FHR_E and FHR_U signal analysis, concerning the long- and short-term variability, calculated using signal in a form of time event series—a sequence of events unevenly localized in time, together with the SMPD values estimating the inconsistencies between both the methods**.

**Index**	**FHR_E**		**FHR_U**		**SMPD (%)**
	**Mean**	**SD**	**Mean**	**SD**	
Haan_LTI	21.89	14.69	20.62	14.59	6
Yeh_II 10^2^	2.89	1.60	2.57	1.53	12
Organ_LTV	8.18	4.46	7.35	4.35	11
Dalton_LTV	12.18	6.68	10.78	6.36	12
Zugaib_LTV 10^2^	2.22	1.25	2.01	1.21	10
Haan_STI 10^3^^#^	6.29	1.94	3.10	1.02	68
Yeh_DI 10^3^^#^	6.08	2.33	3.62	1.30	51
Geijn_STV^*^	15.27	23.70	8.98	13.51	52
Dalton_STV^#^	1.80	0.65	1.01	0.32	56
Zugaib_STV 10^3^^#^	2.77	1.02	1.76	0.58	45
SampEn^+^	1.57	0.57	1.27	0.48	21

* p < 0.03;

+ p < 0.001;

# *p < 0.0001 (paired t-test)*.

With regard to such large inconsistencies it has to be decided which of the two methods may be considered as providing the FHR variability description being closer to the true one. The answer is not obvious, because in this work no reference signal was acquired simultaneously with two analyzed methods. Such gold standard can be provided by previously mentioned the direct fetal electrocardiography, where the pure FECG is acquired from the fetal head. In the previous studies where the FHR signal from ultrasound method was compared with the reference one, it has been shown that ultrasound method underestimates the short-term variability on a level between 20 and 40% in reference to direct fetal electrocardiography. A similar trend can be seen in Table 3, where the FHR_U is compared with FHR_E obtained from the abdominal fetal electrocardiogram.

The signal database has been divided into two groups: normal and abnormal, according to the established fetal outcome criteria. The description of two signal groups is shown in Table 4. Summary of the FHR signal analysis results, comprising 15 clinical parameters determined for both groups of fetal outcome, are shown separately for the ultrasound method (Table 5) and abdominal electrocardiography (Table 5). In addition to the mean value and standard deviation, the mean percentage difference MPD was calculated, assuming the values obtained in normal fetal outcome group as the reference. Apart from assessing the statistical significance of the difference between the normal and abnormal groups, also the tendency of changes was studied for the particular parameters between these groups. It was carried out to check whether the observed tendency would be consistent with the clinical interpretation of those parameters. Considering the number of ACC patterns, a significant difference between the abnormal and normal groups was noted, and the tendency was consistent. But for the number of DEC patterns no significant difference was observed. The MPD took the opposite values: negative value for ultrasound (−48%) and positive for electrocardiography (38%). Thus, a clinical interpretation of decelerations as a sign of fetal distress has been confirmed only in case of electrocardiography. It is mainly caused by the different amount of the signal loss episodes in both types of signal. Particularly, in the FHR_U the signal loss often occurs during the deceleration which leads to misdetection of this pattern (Figure 3). As for the accelerations both methods follow the clinical meaning as they provided a higher number of such episodes relating to fetal well-being in normal group than in abnormal one.

**Table 4:** **Descriptive statistics of the selected parameters, describing the labor and fetal outcome, presented separately for two groups of the research material, including 19 recordings with abnormal and 41 recordings with normal fetal outcome**.

	**Normal (*n* = 41)**	**Abnormal (*n* = 19)**
Gestational age at delivery (weeks)	37.5 (±3.2)	34.0 (±4.8)
Range of gestational age at delivery (weeks)	35–41	26–40
Birth weight of a newborn (g)	3,164 (±576)	2,038 (±777)
Range of birth weight (g)	2,309–4,480	740–3,690
Intrauterine growth restriction (<5th percentile)	0	7
Vaginal delivery (number)	19 (46%)	3 (16%)
Caesarean section (number)	22 (54%)	16 (84%)
NICU stay >24 h (number)	0	8
Apgar score (at 5 min) <7	0	12
pH <7.2	0	9
BE > 12 (mmol/l)	0	6

**Table 5:** **Summary of the FHR-E and FHR-U signal analysis comprising 15 clinical parameters determined using computer-aided fetal monitoring system, for two groups of fetal outcome, together with the mean percentage difference MPD depicting the inconsistencies between both the groups**.

**Parameters**	**FHR_E**					**FHR_U**				
	**Normal**		**Abnormal**		**MPD (%)**	**Normal**		**Abnormal**		**MPD (%)**
	**Mean**	**SD**	**Mean**	**SD**		**Mean**	**SD**	**Mean**	**SD**	
M_FHR (bpm)	142.96	8.21	143.69	11.68	0.5	143.18	8.24	143.61	12.00	0.3
M_BL (bpm)	141.27	8.39	142.64	11.88	1.0	141.45	8.43	142.54	11.96	0.8
ACC (number)	12.97^*^	7.75	8.15^*^	7.13	−37.2	12.13^#^	7.71	7.49^#^	7.92	−38.3
DEC (number)	0.60	1.34	0.83	1.60	38.3	0.50	1.21	0.26	0.64	−47.9
LTV (ms)	40.67	12.27	37.10	10.98	−8.8	39.01	11.74	35.54	10.55	−8.9
STV (ms)	6.39	2.29	6.25	2.73	−2.2	5.84	1.98	5.33	2.11	−8.7
OSC (ms)	13.82	4.01	12.51	3.22	−9.5	13.33	3.90	12.01	3.12	−9.9
L_HE (min)	13.88	12.34	10.21	11.65	−26.4	11.76	11.57	8.11	11.21	−31.1
L_LE (min)	7.02	7.44	9.63	9.07	37.1	7.98	7.82	10.32	8.08	29.3
V_HE (ms)	55.13	8.27	50.07	11.90	−9.2	56.06	7.29	51.78	8.82	−7.6
V_LE (ms)	18.28	3.58	19.44	2.28	6.3	18.11	3.09	20.06	3.70	10.8
OSC_I (%)	11.34	16.37	8.69	8.86	−23.4	11.96	17.15	9.09	10.39	−24.0
OSC_II (%)	25.90	14.97	31.79	16.54	22.7	27.36	14.85	33.46	16.13	22.3
OSC_III (%)	45.41	19.28	45.09	16.09	−0.7	45.61	19.71	45.20	18.27	−0.9
OSC_IV (%)	10.62	10.42	5.37	4.40	−49.4	9.44	10.18	4.28	4.44	−54.6

* p < 0.05;

# *p < 0.05 (t-test)*.

As above, no statistically significant differences were noted for all the indices describing both the short-term and long-term FHR variability. Decrease of most of the long-term variability indices was noted in abnormal groups (MPD ranged from −20 to −9%) for both FHR_U (Table 6) and FHR_E signals (Table 6). This is consistent with the clinical interpretation of these indices, since a decrease of FHR variability is regarded as the fetal distress sign. Also, for most of the short-term variability indices a decrease was noted in the abnormal group. In the case of the US method, the MPD takes value of −12%, but for the FECG method it ranges between smaller values—from −4 to −2%. An interesting property is shown by the Geijn_STV index, which regardless of the acquisition method used, exceeds the mentioned range for short-term variability indices in the abnormal group, as it is significantly reduced (MPD = −36% for both methods). Contrary to the Geijn_STV, the Haan_STI although also exceeds the mentioned range for FHR_E signals, but shows different tendency—it is overestimated by 3%. Finally, it should be noted that the variability indices are capable to differentiate the signals relating to normal and abnormal fetal state, and among them the Geijn_STV index is particularly effective.

**Table 6:** **Results of the FHR_E and FHR_U signal analysis, in terms of the instantaneous FHR variability assessment, calculated for signal in a form of time event series, together with the mean percentage difference MPD depicting the inconsistencies between both the groups**.

**Index**	**FHR_E**					**FHR_U**				
	**Normal**		**Abnormal**		**MPD (%)**	**Normal**		**Abnormal**		**MPD (%)**
	**Mean**	**SD**	**Mean**	**SD**		**Mean**	**SD**	**Mean**	**SD**	
Haan_LTI	22.76	15.35	19.87	13.14	−13	21.9	15.55	17.63	12.33	−19
Yeh_II 10^2^	2.99	1.64	2.66	1.49	−11	2.7	1.58	2.25	1.40	−17
Organ_LTV	8.49	4.63	7.48	4.07	−12	7.75	4.56	6.39	3.86	−18
Dalton_LTV	12.58	6.84	11.24	6.29	−11	11.33	6.55	9.48	5.91	−16
Zugaib_LTV 10^2^	2.29	1.30	2.04	1.14	−11	2.12	1.27	1.75	1.07	−17
Haan_STI 10^3^	6.24	1.96	6.43	1.87	3	3.22	1.07	2.83	0.91	−12
Yeh_DI 10^3^	6.15	2.33	5.92	2.34	−4	3.76	1.34	3.31	1.20	−12
Geijn_STV	17.1	31.96	10.99	4.36	−36	10.05	18.17	6.48	2.62	−36
Dalton_STV	1.81	0.66	1.77	0.64	−2	1.05	0.34	0.92	0.29	−12
Zugaib_STV 10^3^	2.8	1.03	2.68	0.99	−4	1.82	0.60	1.6	0.53	−12
SampEn_FHR	1.61	0.68	1.53	0.56	−5	1.29	0.55	1.24	0.45	−5

## Conclusions

The paper proposes an extended process of comparing two different methods of fetal heart rate monitoring. It takes into account the issues associated with comparing different biomedical signals—the unsatisfying usability of the results obtained from direct signal comparison. High reliability of the comparison results can only be ensured by using the clinically significant parameters determined for signals acquired by both methods being analyzed (Jezewski et al., 2006). Initial preparation of the research material has been stated as very important step too, in order to ensure the results are not affected with different measurement conditions. Two different methods of measuring the fetal heart rate signal were analyzed: indirect electrocardiography for recording the electrical heart activity from maternal abdominal wall, and the pulsed Doppler ultrasound method based on mechanical activity of the fetal heart (Cohen et al., 2012). None of these methods can be considered as a reference, due to a number of measurement error sources identified (Goncalves et al., 2015).

There is a strong conviction that the ultrasound method, as leading in clinical practice, can serve as a quasi-reference. It applies when the acquired fetal heart rate signal is interpreted both visually and more and more often, using the results of quantitative analysis in the computer-aided fetal monitoring system. On the other hand, we observe growing expectations on the indirect fetal electrocardiography, especially with regard to its application for pregnancy telemonitoring at home (Martinek et al., 2015). The above issues justified a need for the comparison between electrocardiography and ultrasound method presented in this paper in a view of usability of the results obtained. The complex process of data synchronization and validation within the research material resulted in 60 pairs of FHR signals, with an average duration of about 33 min. Obtained low level of the signal loss (4.5% for the US and 1.8% for FECG method) enabled to perform both direct comparison and indirect one—by using clinically relevant parameters (Reinhard et al., 2010; Wrobel et al., 2015b).

From direct comparison it has been resulted there is no measurement bias between the acquisition methods. The mean difference between the FHR_E and FHR_U signal was equal to −0.2 bpm (*SD* = 0.38 bpm). On the other hand, the mean absolute difference measured between the methods, being important for both visual and computer-aided signal analysis, was equal to 1.2 bpm. When relating to typical FHR level of about 140 bpm, the inconsistency takes the value of about 0.9%. These results are similar to those obtained in Cohen et al. (2012) where the ultrasound method was compared with the reference direct fetal electrocardiography and in Jezewski et al. (2012), where abdominal electrocardiography was compared with reference in a similar manner.

Such low differences do not affect the visual assessment of the FHR signal, taking into account a resolution limit of both a printer and human eye (Reinhard et al., 2012). However, when analyzing the results of the indirect comparison, by using the parameters quantitatively describing the clinical features, the inconsistencies of several percent were noted. This particularly affects the patterns being sensitive to the instantaneous differences in FHR values, like acceleration (7.8%) and particularly deceleration (54%), where (for the ultrasound method) the signal loss within the episodes has a significant impact (Voicu et al., 2010). Similarly, significant differences were noted between the ultrasound method and the reference direct electrocardiography in Desai et al. (2013).

The results obtained for the long- and short-term FHR variability indices show significant overestimation of their values in the signal acquired using the electrocardiography, by 10% and 50%, respectively. However, the results of work (Jezewski et al., 2006), where the US method was related to the reference direct FECG, have shown that the electrical method provides significantly higher FHR variability. Hence it leads to conclusion, that the variability indices for the FECG signal acquired from the abdominal wall represent the true FHR variability, whereas in the ultrasound method they are significantly underestimated (Goncalves et al., 2013). On the other hand, a comparison of these methods, through a clinical interpretation of the FHR signals for fetal outcome prediction was examined. It showed that ability of various clinical parameters to distinguish between normal and abnormal groups do not depend on the acquisition method. That was confirmed by similar tendency of changes of clinical parameters determined in both groups.

In summary we can conclude that the abdominal fetal electrocardiography, being considered as an alternative to the ultrasound based approach for certain application, like a pregnancy telemonitoring at home, does not change significantly the interpretation of the FHR signal. Equivalence of these methods was confirmed for both visual assessment and automated analysis of the signals. Despite the lack of reference signal, it can be proved indirectly that the abdominal fetal electrocardiography provides more reliable description of the instantaneous FHR variability. It's another advantage over the ultrasound method relates to a lower signal loss. However, this conclusion coming from analysis of the signals collected in hospital conditions may undergo final verification when both methods will become widely applied in the systems for pregnancy telemonitoring.

## Ethics statement

The study protocol was approved by the Ethical Committee of the Silesian Medical University, Katowice, Poland (NN-013-123/03). Subjects read approved consent form and gave written informed consent to participate in the study.

## Author contributions

JJ, JW, and AM designed the study approach and experiments. KH and AM described the research background in the introduction section. KH and JJ collected the research material. TK and RM provided the signal synchronization procedures. JJ, JW, and MJ were responsible for analysis and interpretation of data. TK and JJ performed statistics. JW wrote the manuscript with contributions from JJ, KH, RM, and MJ. All authors read and approved the final manuscript.

### Conflict of interest statement

The authors declare that the research was conducted in the absence of any commercial or financial relationships that could be construed as a potential conflict of interest. The reviewer HA and handling Editor declared their shared affiliation, and the handling Editor states that the process nevertheless met the standards of a fair and objective review.
